# Probing the Cytoadherence of Malaria Infected Red Blood Cells under Flow

**DOI:** 10.1371/journal.pone.0064763

**Published:** 2013-05-28

**Authors:** Xiaofeng Xu, Artem K. Efremov, Ang Li, Lipeng Lai, Ming Dao, Chwee Teck Lim, Jianshu Cao

**Affiliations:** 1 Singapore-MIT Alliance for Research and Technology (SMART), Singapore, Singapore; 2 NUS Graduate School for Integrative Sciences and Engineering, National University of Singapore, Singapore, Singapore; 3 Department of Chemistry, Massachusetts Institute of Technology, Cambridge, Massachusetts, United States of America; 4 MIT-SUTD Collaboration, Massachusetts Institute of Technology, Cambridge, Massachusetts, United States of America; 5 Department of Material Science and Engineering, Massachusetts Institute of Technology, Cambridge, Massachusetts, United States of America; 6 Nano Biomechanics Laboratory, Department of Bioengineering and Department of Mechanical Engineering, National University of Singapore, Singapore, Singapore; 7 Mechanobiology Institute, National University of Singapore, Singapore, Singapore; Université Pierre et Marie Curie, France

## Abstract

Malaria is one of the most widespread and deadly human parasitic diseases caused by the *Plasmodium (P.)* species with the *P.falciparum* being the most deadly. The parasites are capable of invading red blood cells (RBCs) during infection. At the late stage of parasites’ development, the parasites export proteins to the infected RBCs (iRBC) membrane and bind to receptors of surface proteins on the endothelial cells that line microvasculature walls. Resulting adhesion of iRBCs to microvasculature is one of the main sources of most complications during malaria infection. Therefore, it is important to develop a versatile and simple experimental method to quantitatively investigate iRBCs cytoadhesion and binding kinetics. Here, we developed an advanced flow based adhesion assay to demonstrate that iRBC’s adhesion to endothelial CD36 receptor protein coated channels is a bistable process possessing a hysteresis loop. This finding confirms a recently developed model of cell adhesion which we used to fit our experimental data. We measured the contact area of iRBC under shear flow at different stages of infection using Total Internal Reflection Fluorescence (TIRF), and also adhesion receptor and ligand binding kinetics using Atomic Force Microscopy (AFM). With these parameters, we reproduced in our model the experimentally observed changes in adhesion properties of iRBCs accompanying parasite maturation and investigated the main mechanisms responsible for these changes, which are the contact area during the shear flow as well as the rupture area size.

## Introduction

Malaria is a mosquito-borne parasitic disease caused by the eukaryotic protists of the genus *Plasmodium*. There are five species of the parasite that affect humans, *P. falciparum, P. vivax, P. ovale, P. malariae* and *P. knowlesi. P. falciparum* is the most deadly and is predominant in Africa. The latest report from WHO in 2011 shows that there were an estimated 216 million episodes of malaria worldwide in 2010 from which ∼ 655,000 resulted in death. Approximately 86% of malaria deaths were in children under the age of five [1].

When a mosquito injects motile sporozoites into the human blood stream during a blood meal, they will invade the liver cells where they will multiply and generate thousands of merozoites. These merozoites are then released into the blood stream and start to invade healthy RBCs. The infected RBCs (iRBCs) go through a sequence of three main developmental stages – ring, trophozoite and schizont, also known as the asexual multiplication cycle. After 48 hours this sequence ends in the bursting and releasing of 16–32 new merozoites [2] from the iRBCs, and the cycle of invasion and infection starts again. During this cycle, iRBCs circulating in the blood stream begin to lose their deformability, thus becoming potential targets for filtering and destruction by the spleen. To avoid this, the parasite expresses and exports adhesive proteins to the surface of the host iRBC, causing the cell to stick to microvascular endothelial cells in different organs, thereby preventing its clearance by the spleen. This adhesion is believed to be one of the main causes of lethal complications resulting in cerebral malaria and placental malaria.

Existing experimental data suggests that iRBC cytoadhesion is mostly mediated by the *P. falciparum* erythrocyte membrane protein 1 (PfEMP1) and microvascular endothelium cell receptors, such as CD36, intercellular adhesion molecule 1 (ICAM-1) and thrombospondin (TSP) [3]. However, although some of the major receptors participating in the iRBC adhesion and their kinetic parameters have been obtained from single molecule force spectroscopy experiments using atomic force microscope (AFM) [4], complete understanding of the iRBC adhesion kinetics is still unclear since the cell adhesion process involves other important factors such as receptor binding rate, contact and rupture area size, etc, which were not previously measured for iRBCs.

Here, we describe an advanced experimental flow adhesion assay that comprises a microfluidic channel with controllable flow rate mounted on a Total Internal Reflection Microscopy (TIRF) microscope. We also performed atomic force microscopy (AFM) probing of CD36-PfEMP1 interactions to calculate the binding kinetics of iRBCs to CD36 protein and compare with results obtained from flow adhesion tests performed at different stages of parasite development. Finally, our experimental results confirm the prediction of a theoretical model [5].

## Materials and Methods

### Ethics Statement

The present study was approved by the National University of Singapore (NUS) institutional review board. The participants provided their written consent to participate in this study.

### Parasite Culture

In this study, we used *Plasmodium falciparum* 3D7 knob positive [4]. Parasites were cultured *in vitro* according to a conventional protocol [6]. Parasites were grown in human erythrocytes in RPMI 1640 medium (Invitrogen) supplemented with 0.5% Albumax I (Invitrogen), 2 mM L-glutamine, 50 µg/ml hypoxanthine and 25 µg/ml gentamycin at 2.5% hematocrit and cultured at 5% CO_2_, 3% O_2_ and 92% N_2_ at 37°C. When the majority of parasites were in the ring stage, synchronization by using 5% sorbitol was conducted twice with 6–8 hours interval in order to obtain trophozoite stage iRBCs in 12 hours and schizont stage iRBCs in 24 hours after synchronization. Experimentally measured parasitemia in each experiment was greater than 3%.

### Microfluidic Channel Preparation

**Figure 1:**
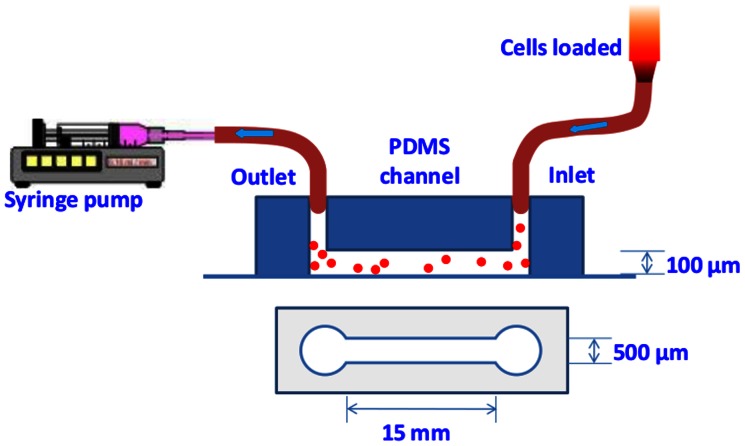
Schematic of microfluidic flow-based adhesion experiment setup. Cells are loaded from inlet side and flow through the microfluidic PDMS channel. The shear stress inside the channel is controlled by the flow rate of syringe pump.

In our flow adhesion experiments we used a custom-made microfluidic PDMS channel with dimensions of 500 µm (width) by 100 µm (depth) by 1.5 cm (length) and with two punched holes for inlet and outlet (Figure 1). The PDMS was permanently bounded to a glass slide by plasma treatment (Harrick Plasma). In each experiment, the channel was prewashed with 70% alcohol followed by distilled water washing. The cleaned channel was then filled with 20 µl human CD36 protein (Sino Biological Inc) with concentration of interest, 20 µg/ml and 50 µg/ml (see Results and discussion section) and incubated for overnight at 4°C. Before the flow experiment, CD36 coated channel was washed with phosphate buffered saline (PBS) (Invitrogen) and then coated with 1–3% bovine serum albumin (BSA) (Millenyi Biotec) in PBS and incubated for 30 min in this solution at room temperature to block nonspecific binding of iRBCs to the channel. After the final wash with PBS, the flow chamber was mounted on the Olympus X71 microscope stage with plastic tape. Inlet and outlet holes of the channel were connected to the loading reservoir and syringe by Tygon® tubing, respectively. IRBCs were loaded into the channel from an open syringe by withdrawing the syringe pump and thus, generating a negative pressure inside the channel, as shown in Figure 1. Resulting shear stress created in the channel was controlled by the flow rate of the syringe pump [7] (New Era Pump Systems, Inc.). In our experiments, applied pressure was varied from 0.1 Pa to several Pa, in increasing and decreasing steps of 0.1 Pa, 0.3 Pa, 0.5 Pa, 0.7 Pa, 1 Pa, 1.2 Pa, 1.5 Pa (See Result and discussion – Flow experiments section). At each step, the shear stress was maintained for 5 min in order to achieve steady-state flow and five images were taken from different fields of view using a 20X objective. Based on these pictures, the number of cells adhering to the surface of the channel was calculated and the resulting detachment curves were plotted as functions of the shear rate (see Results and discussion section). Each experiment was repeated more than three times for each of the different parasite stages as well as different coatings of CD36 protein concentration.

### Total Internal Reflection Fluorescence (TIRF) Experiments

To visualize and measure the contact area between iRBCs and the surface of the channel, we applied TIRF microscopy to cells stained with a fluorescent dye, as described below. Before each TIRF experiment, we used Magnetic-activated cell sorting (MACS) method to enrich the trophozoite or the schizont stage infected RBCs from initial culture [8]. After enrichment, the fraction of iRBCs at the trophozoite/schizont stage reached more than 90% by checking the Giemsa stained smear glass slide of enriched sample under microscope. Enriched iRBCs were then stained using 5 µg/ml lipophilic styryl dye, FM® 1–43 dye (Invitrogen), which has no effect on the iRBC’s deformability (see Text S1), for half an hour, followed by washing with warm RPMI or malaria culture medium for at least 4–6 times. Finally, fresh healthy RBCs as well as malaria culture medium were added in order to reach 2.5% hematocrit and 3–4% parasitemia similar to the condition of the flow experiments described above.

**Figure 2:**
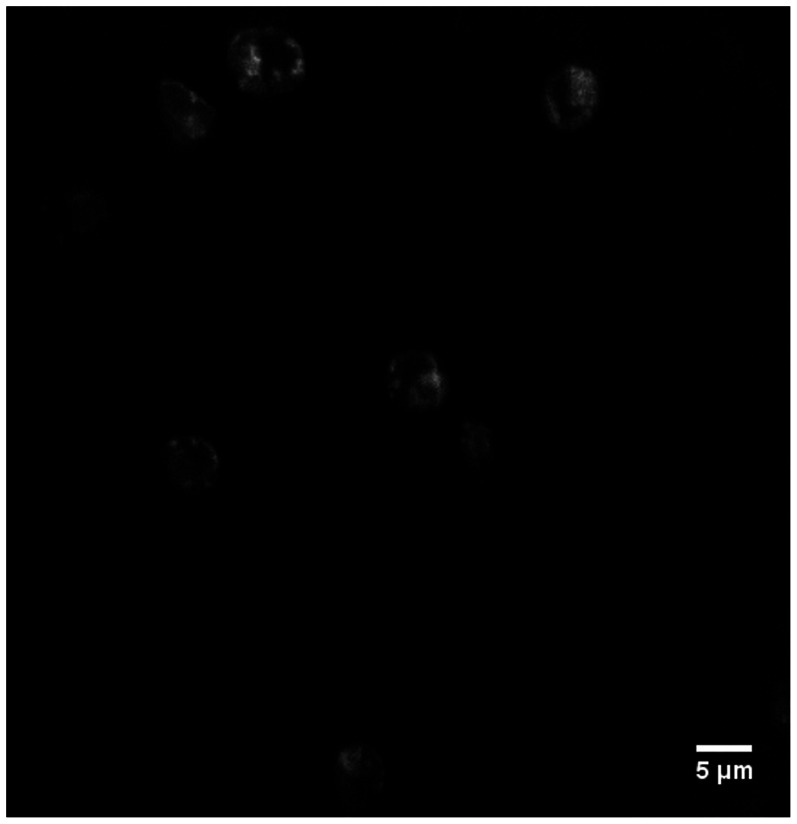
TIRF image of FM®1–43 stained iRBCs attached to the CD36 coated channel.

TIRF experiments were carried out on Olympus IX71 using 100X objective and 488 nm laser. The FM®1–43 stained iRBC combined with fresh healthy RBCs were injected into the CD36 protein coated channel using the same flow experiment setup as described above. After the cells had attached to the surface of the channel, the contact area between iRBCs and the channel was visualized by TIRF, which excites only fluorophores residing in the restricted region of the membrane-glass interface (Figure 2). In each experiment we collected more than 10 images of the contact areas from different fields of view at each shear stress using a homemade program on GIOR controlling the camera functions. To gather enough data for statistical analysis, each experiment was repeated more than three times. Acquired images were then processed using ImageJ program, and the contact areas between iRBCs and the channel surface were calculated based on the pixel size measurements (each pixel = 160 nm).

### Atomic Force Microscopy (AFM) Measurements

To measure unstrained detachment rate of CD36-PfEMP1 bonds and their compliance, we used the same AFM experimental setup as is described by Li A. et al. [4]. The CD36 protein was immobilized on the AFM tip via steps of covalent bonding and iRBCs were immobilized on glass substrate via strong interaction between PHA-E lectin and glycoprotein on the iRBC surface.

### Theoretical Model

**Figure 3:**
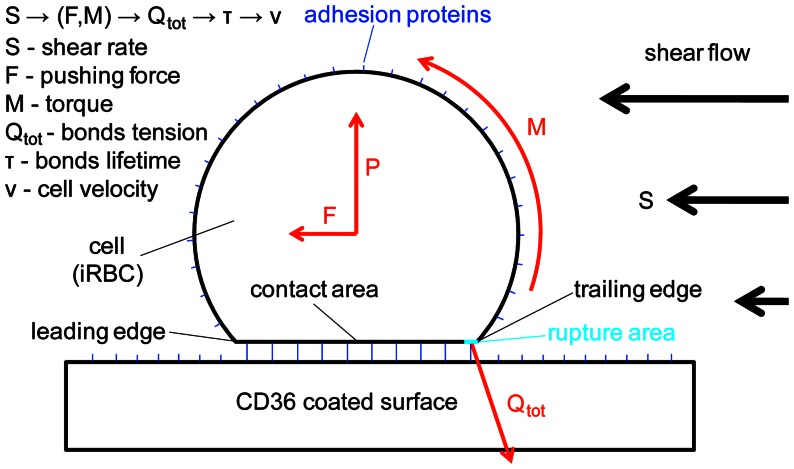
Mathematical model of cell adhesion in shear flow [5]. An iRBC rolling on the CD36 coated surface is shown. Media flow characterized by the shear rate (*S*) generates a force (*F*) and torque (*M*) on the cell. These force and torque are balanced by the bonds tension (*Q*) in the rupture area and the normal reaction (*P*) acting on the cell so that the cell at every instant is in mechanical quasi-equilibrium. The bond tension determines the average bond lifetime (τ) in the rupture area, which, in turn, is closely connected to the average velocity of the cell (*v* ≈ *c*/τ, where c is the rupture area size). Reprinted with permission from Elsevier.

**Figure 4:**
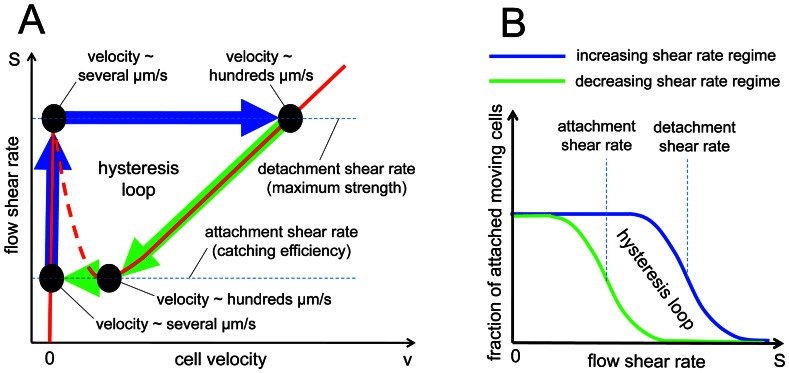
Cell adhesion bistability in shear flow [5]. (A) A stochastic model from ref. [5] predicts that cell adhesion is a bistable process in shear flow, which manifests itself as a hysteresis loop in shear stress-velocity coordinates. (B) The shear stress-velocity hysteresis loop results in an attached cells-shear rate loop, which can be measured experimentally using flow based adhesion assay developed in this paper. Reprinted with permission from Elsevier.

To fit experimental data we used a mathematical model recently-developed by *Efremov and Cao* [5]. In this stochastic model a cell interacting with adhesion proteins on blood-vessel walls is represented by a sphere (with radius *r*), which rolls on a planar surface under the load *F* and torque *M* created by the shear flow (see Figure 3). During cell rolling, new bonds between the cell and the surface are continuously formed in the contact area, which is characterized by the mean width *a*. At the same time, previously formed bonds rupture at the trailing edge of the contact area due to the tension *Q_tot_* generated by the load *F* and torque *M*. We will call this region– rupture area [5], characterized by its size *c*. Solving hydrodynamic and the force balance equations (see Text S2) of the cell motion one can establish a relationship between the shear stress of the flow *S* and the rupture force *Q_tot_* acting on the bonds in the rupture area. Given the rupture force and the adhesion proteins unstressed kinetic rates for bonds formation *k*_+_ and dissociation *k_off_* one can estimate the average concentration and lifetime of adhesion bonds in the rupture area and thus, find the average rolling velocity of the cell, *v*. The resulting equation between the shear stress and the cell velocity (eq. 12 in the Efremov and Cao’s paper [5]) shows that cell adhesion is a bistable process possessing a hysteresis loop (see Figure 4A). The shear stress-velocity hysteresis loop leads to another hysteresis loop of shear stress-normalized number of attached cell (see Figure 4B), which can be measured directly in the flow experiments (see Results and discussion – flow experiment section). Thus, experimentally measuring (*i*) the shear stress-normalized number of attached cell hysteresis loop of iRBCs attached to the CD36 coated channel at different parasite development stages, (*ii*) the shear stress, *S*, (*iii*) medium viscosity, *η*, (*iv*) cells average radius, *r*, (*v*) contact area width distribution, *a ± δa*, (*vi*) unstrained bonds rupture rate *k_off_* and binding potential width *x* ^#^, and then using these numbers as input parameters for the stochastic model, we determined how relevant adhesion properties of iRBCs (namely the average rupture area size *c* and receptors diffusive binding rate *k*_+_) change at different stages of the parasite maturation (see Results and discussion section for more details).

### Statistical Analysis

The data was reported as mean ± standard error of mean (SEM). Analyses of multiple groups were performed with one-way ANOVA and Tukey test. Student t-test was used for comparison of two groups. The level of significance was calculated with *p* value less than 0.05.

## Results and Discussion

### The Contact Area Measurement

**Figure 5:**
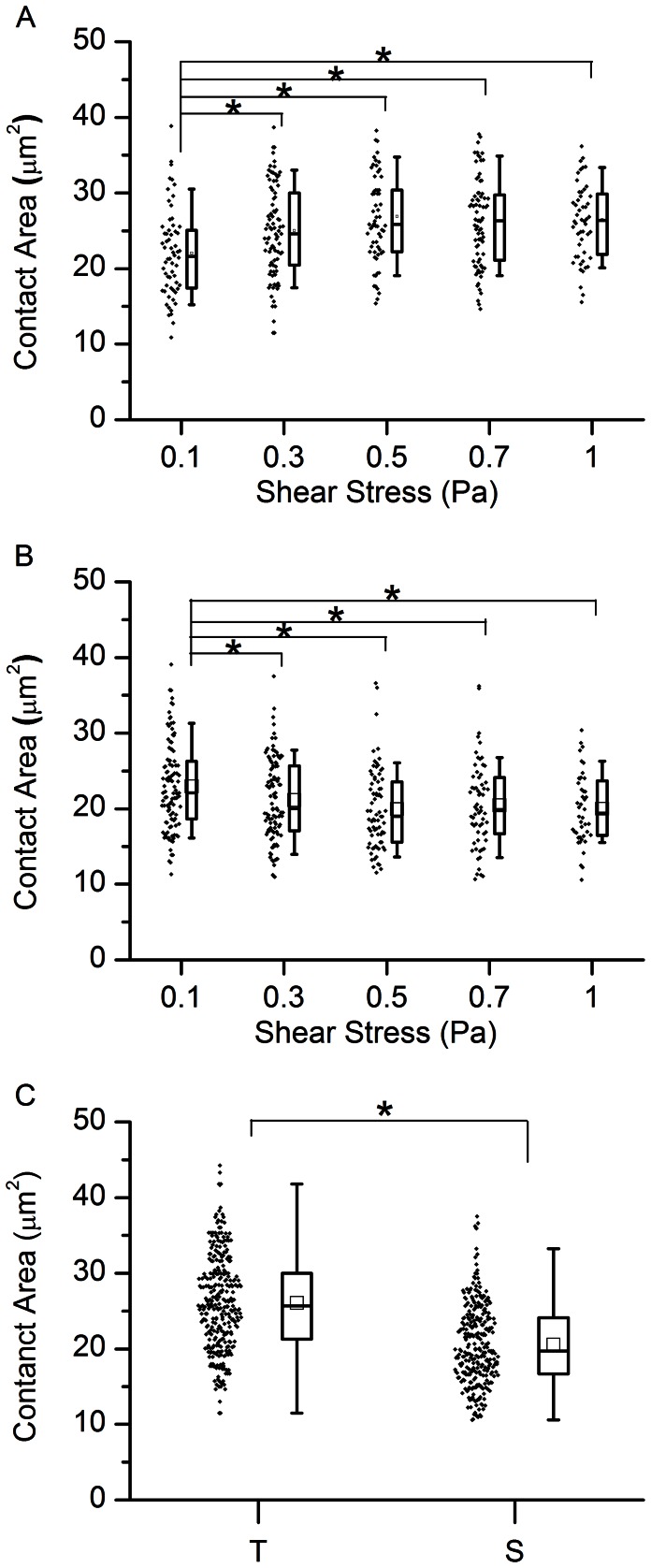
Measurements of contact area of iRBCs flowing through CD36 coated channel using TIRF. (A) Box plot of the contact area of iRBCs at the trophozoite stage flowing through the CD36 coated channel at different shear stresses. The bottom and top of the box denote the 25^th^ and 75^th^ percentiles of the population, respectively, while the bottom and top whiskers denote 10^th^ and 90^th^ percentiles, respectively. (B) The same as that in panel A only for iRBCs at the schizont stage. There is no significant difference among groups, where shear stress larger than 0.3 Pa. (C) The same as in panel A for contact area of iRBC at the trophozoite stage and the schizont stage at shear stress larger than 0.3 Pa. *indicates significant difference at *p*<0.05.

Recently reported mathematical models of cell adhesion [5], [9], [10]suggest that the strength of cell adhesion can strongly depend on the size of the contact area between a cell and the surface with which it interacts. Thus, characterization of iRBCs interaction with the CD36 coated surface at different stages of parasite development requires knowledge about the size of the contact area at these stages. To measure it, we performed a TIRF-based assay at different shear stresses for iRBCs at the trophozoite and the schizont stages, as described in the Methods section. Our experimental results show that the average contact area at the trophozoite stage (25.36±6.24 µm^2^) is larger than the average contact area at the schizont stage (21.20±5.55 µm^2^) (see Figure 5C), and in both cases, it is weakly dependent on the flow shear stress (see Figure 5A and 5B). Firstly, from statistical analysis for shear stress larger than 0.3 Pa, the contact areas are not significantly different at the trophozoite or schizont stage (*p*>0.05), whereas at 0.1 Pa, the mean of contact area is significantly different when compared to higher shear stress at both the trophozoite and the schizont stages. This is because when iRBCs flow in the microfluidic channel, the 0.1 Pa shear stress is not high enough to cause any appreciable deformation between the two iRBC stages. When shear stress is larger than 0.3 Pa, the trophozoite stage iRBCs which are more deformable than the schizont stage iRBCs, the contact area increases, but remains relatively constant with further increase of the shear stress. At the schizont stage, the contact area decreased at 0.3 Pa, but remained relatively constant at higher shear stresses. Since the average contact area did not change much for shear stresses larger than 0.3 Pa at both the schizont and the trophozoite stages, we assumed that it is constant in our model. Secondly, comparing the trophozoite stage and the schizont stage for contact area at shear stresses larger than 0.3 Pa, the *p*-values were smaller than 0.05, which means they were significantly different (Figure 5C). Both of these observations agree well with existing experimental and theoretical data: due to the growing parasites inside the iRBCs, their volume to surface ratio increased with time to the one of a sphere. As a result, effective deformability of iRBCs decreases as they enter into the late stages of parasite development since cells with higher volume to surface ratio are generally less deformable [11]. According to the existing mathematical models of cell adhesion [9], the cell-surface contact area becomes smaller as the cell deformability decreases. Additionally, dependence of the contact area on the flow shear rate weakens with decreasing cell deformability. Since the stiffness of iRBCs at the schizont stage is higher than that at the trophozoite stage, our results (shown on Figure 5A and B) qualitatively agree with these theoretical predictions.

### PfEMP1-CD36 Bonds Dissociation Rate

**Figure 6:**
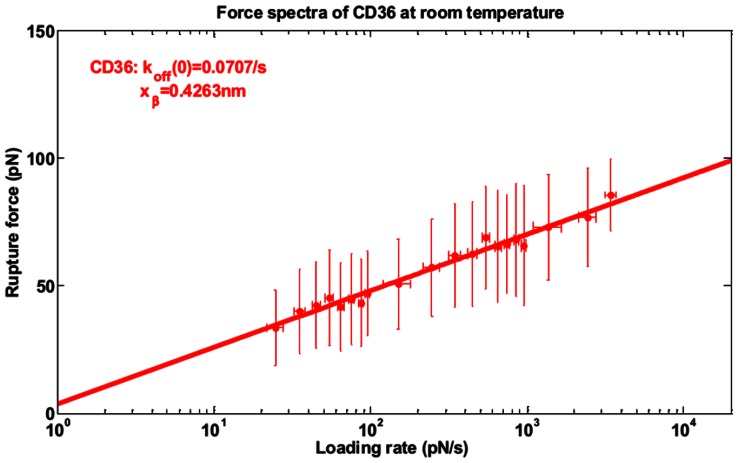
Force spectrum and the extrapolated kinetic parameter of CD36-iRBCs bonds at room temperature. Mean rupture forces within each binned window of loading rates were used as pooled measures to fit to the Bell-Evan’s model for the characteristic dynamic force spectra reconstruction.

To reduce the number of unknown variables in our model, we measured the unstrained detachment rate and compliance of PfEMP1-CD36 bonds using the same AFM setup as in *Li at al.* [4]. Since the flow experiments we performed were at room temperature, the AFM measurements were also carried out at room temperature in order to be consistent with the flow experiments. Resulting force spectrum is shown in Figure 6. The Bell-Evans’ model [12], [13] fitting to this spectrum gives the unstrained mean dissociation rate, *k_off_* = 0.707 s^−1^, and the bonds compliance, x^#^ = 0.43 nm.

### Flow Experiments

**Figure 7:**
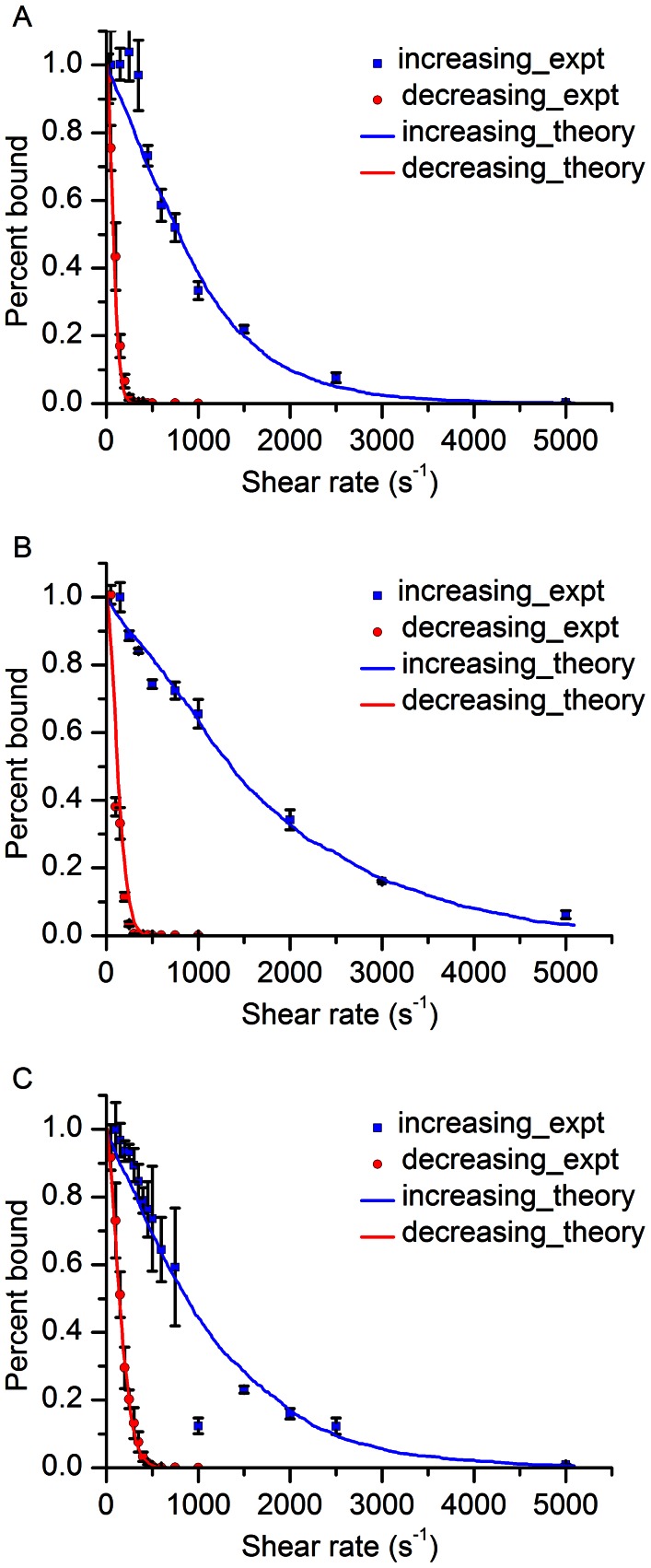
Experimental observation of the cytoadhesion hysteresis. (A-C) Experimental data points and their theoretical fitting are shown. (A) and (B) depict experimental / theoretical results which were obtained for iRBCs at the trophozoite stage flowing through the channel coated with CD36 proteins at concentrations of 20 and 50 µg/ml, respectively (C) shows the results for iRBCs at the schizont stage flowing through the channel coated with CD36 proteins at concentration of 50 µg/ml.

In order to check the model prediction of cell adhesion bistability in shear flow [5], we counted the number of iRBCs attached to the CD36 coated surface at different shear stresses. The latter was changed in increasing and decreasing stepwise manner from 0.1 Pa to 5 Pa and 5 Pa to 0.1 Pa, respectively, because cell adhesion model [5] suggests that the cell adhesion bistability would result in a different behavior of the shear stress-number of attached cell curves measured within these two increasing and decreasing shear stress regions. The cell adhesion bistability can be interpreted as a system memory about past actions, which leads to an appearance of a hysteresis loop between the two curves mentioned above. Indeed, flow experiments performed on the trophozoite stage iRBCs at two different concentrations of CD36 (20 and 50 µg/ml) as well as on the schizont stage iRBCs (at CD36 concentration of 50 µg/ml) showed presence of such a hysteresis loop, see Figure7A–C. Thus, our experimental results confirm the theoretical prediction of the iRBC cytoadhesion bistability in shear flow. As can be seen from Figure 7A and B, at a higher concentration of CD36 receptors on the flow channel surface, the hysteresis loop was larger and adherent trophozoite iRBCs could withstand larger flow shear stresses (when the shear stress was increased from a small value to a high one), which is not surprising since the higher concentration of CD36 leads to generation of a larger number of bonds between iRBCs and the CD36 coated surface. But what is unusual in these results is how the hysteresis loop and iRBCs adhesion strength changes from the trophozoite to the schizont stage. From existing experimental data, it is known that the surface concentration of knobs, special protein clusters on the surface of iRBCs that mediate iRBC cytoadhesion, in the schizont stage is ∼1.4 times higher than in the trophozoite stage (∼7 knobs per µm^2^ in the schizont stage and ∼5 knobs per µm^2^ in the trophozoite stage) [14]. Thus, one would expect that the strength of iRBCs adhesion in the schizont stage is higher than that in the trophozoite stage. However, our experimental data suggests that this is not the case – at the same concentration of CD36 the hysteresis loop in the trophozoite stage is larger than that in the schizont stage suggesting that iRBCs attached to the CD36 coated surface of the flow chamber more strongly in the trophozoite stage than in the schizont stage (see Figure 7B and C). This effect can be explained by a smaller contact area at the schizont stage since the average contact area at the trophozoite and schizont stages in Figure 5A and B are significantly different at shear stresses larger than 0.3 Pa (See Figure 5C).

### Fitting of Experimental Data with Bistability Model

To gain further insights into the iRBC adhesion process and to find the mechanism leading to the decrease of the iRBCs adhesion strength in the schizont stage (i.e. the hysteresis loop area is smaller compared to the trophozoite stage at the same protein concentration), we fitted the experimental data depicted on Figure 7A–C using the bistability model [5]. Experimentally measured model parameters (such as the average radius of iRBCs, average contact area and its standard deviation, relative CD36 receptors surface concentration (protein concentration used for microfluidic channel incubation), media viscosity (η = 1.5 mPa.s), bonds unstrained detachment rate and compliance, etc.) are treated as constants in the model. As mentioned in the TIRF measurement of cell contact area, the 20% decrease in contact area at the schizont stage might contribute to the decrease in adhesion strength. Whereas, remaining four unknown model parameters (CD36 receptors absolute surface concentration (numbers of proteins on the microfluidic channel), diffusive binding rate of adhesion proteins (*k*_+_), rupture area size (*c*) and the average number of PfEMP1 proteins per single knob (*N*)) were adjusted to achieve the best fit (Table 1). As can be seen from Figure 7A–C, the resulting theoretical curves fit the experimental data well.

**Table 1:** Values of the parameters used in Figure 7A–C model fitting.

Parameter	Unit	Figure 7A	Figure 7B	Figure 7C
*a*, Length of the contact area	µm	5.0±2.2	5.0±2.2	4.5±2.3
*b*, Width of the contact area	µm	5.0±2.2	5.0±2.2	4.5±2.3
*c*, Length of the rupture area	nm	300	300	140
*r*, Cell radius	µm	4	4	4
*δ,* Size of the gap between the cell and the coverslip	nm	50	50	50
*x^#^*, Binding potential width	nm	0.43	0.43	0.43
*σ_1_,* PfEMP1 density	µm^−2^	35	35	50
*σ_2_,* CD36 density	µm^−2^	20	50	50
*η,* Viscosity	Pa×s	0.0015	0.0015	0.0015
*T*, Temperature	K	300	300	300
*k_+_*, Receptors diffusive binding rate	µm^−2^	0.06	0.06	0.15
*k_off_*, Dissociation rate	s^−1^	0.07	0.07	0.07
*k_on_*, Association rate in the cluster	s^−1^	10	10	10
*N*, Proteins cluster size		7	7	7

The fitting of our experimental data with the model suggests that the average number of PfEMP1 proteins per a single knob does not change much from trophozoite to schizont stage and remains in the range of *N* = 6–8. It should be noted that the amount of knobs on the surface of iRBCs measured in experiments [14] increases by ∼ 40% as well as the total number of PfEMP1 receptors from the trophozoite to the schizont stage, as mentioned before. Thus according to our analysis, newly synthesized PfEMP1 proteins tend to form new knobs on the iRBCs surface rather than to incorporate in old ones. However, an alternative explanation is also possible: PfEMP1 receptors may form large clusters on the knobs, but due to their high local concentration only 6–8 of these receptors on each knob are able to find binding partners on the surface of the flow channel. In any case, the average number of the receptors interacting with the CD36 coated surface per single knob does not seem to be the main factor responsible for the decrease in the schizont iRBCs adhesion strength because it has the same value in the trophozoite and schizont stages.

As for the remaining two model parameters, the diffusive binding rate of the adhesion proteins (*k*_+_) and rupture area size (*c*), the model showed that *k*_+_ increases 2.5 times (from 0.06 to 0.15 µm^2^/s), whereas, *c* decreases ∼ 2 times (from 300 to 140 nm) during iRBCs transition from the trophozoite to the schizont stage. These theoretical results were consistent with existing experimental data. Recently it was shown that the spectrin network of iRBCs degradates significantly in the schizont stage [15], and from experiments it was known that membrane receptors interacting with underlying cytoskeletal network decreases their mobility, see for example [16]. Thus, the more degraded the spectrin network is, the more diffusive are membrane receptors on the iRBC surface, and the faster they bind to their ligands. This explains higher receptor binding rate, *k*_+_, in the schizont stage. On the other hand, at the schizont stage, the network degradation led to less efficient mechano-transduction of the rupture load, *Q_tot_*, acting in the rupture area to other PfEMP1 receptors. Therefore at the schizont stage, the bonds in the rupture area feel the rupture load, and they are “isolated” due to the degradation of spectrin network, once reach the breaking point, the bonds break together, form a smaller rupture area. Whereas for the trophozoite stage cell, firstly the bonds in the rupture area feel the rupture load, but instead of breaking immediately, they transfer the load to the nearby bonds, due to the better connected network. Larger area shares the rupture load, leads to larger rupture area. Moreover, the considerably smaller contact area might also be the cause of smaller rupture area at the schizont stage. As a result, the effective size of the rupture area, *c*, in the schizont stage is smaller. From these observations, it follows that the strength of iRBCs cytoadhesion is strongly dependent on the rupture area size since neither increased amount of adhesion proteins on the iRBCs surface nor their increased binding rate could compensate approximately 2 times decrease of the rupture area in the schizont stage. Indeed, the size of the rupture area determines how many adhesion bonds share the load *Q_tot_*. The higher this number, the smaller is the average force, *Q*, acting on each bond in the rupture area and the larger is the average lifetime, *τ*, of each bond. According to the Bell-Evans model [12], [13], this is an exponential effect since *τ* = *k_off_* ×exp(x^#^ *Q*/k_B_T), where x^#^ is the binding potential width. Thus, even a small decrease of the rupture area size can lead to a considerable decrease in the cell adhesion strength.

With the combined theoretical and experimental studies of cell adhesion under shear flow, we found that the main contributing factors for the change in adhesion strength during parasite maturation are the contact area and the size of the rupture area. Although this is limited to the *in vitro* study of cell protein interactions, it allows us to investigate the binding kinetics of iRBCs to any selected endothelial receptor under shear flow. It can potentially be used to study the efficacy of malaria therapeutics targeting at cytoadhesion. Further research on the flow experimental design and theoretical model will be needed to enable us to better bridge our *in vitro* study to that of *in vivo* physiological conditions.

### Conclusions

Here, we report an advanced microfluidic-flow based assay, which confirms the bistability of the cell adhesion in shear flow, theoretically predicted by a simple stochastic model [5]. Using a combined experimental/theoretical approach, we found that the strength of iRBCs adhesion in the schizont stage is smaller than in the trophozoite stage despite the fact that the surface concentration of adhesion proteins and their binding rate to ligands are higher in the schizont stage. Further analysis allowed us to identify the main factor responsible for the decrease of adhesion strength – the rupture area size, which determines how many adhesion bonds share the rupture load generated by shear flow. To conclude, our advanced flow based assay when combined with the theoretical model of cell adhesion under shear flow can be a useful tool for detailed investigation of the iRBC adhesion process, and can also be extended to other cell types.

## Supporting Information

Text S1 **Micropipette aspiration experiment.** Micropipette aspiration were used to study any possible effects of liphophilic styryl dye, FM® 1–43 dye (Invitrogen) staining on the deformability of iRBCs.
(DOCX)

Text S2 **More detail about model description.** (A) Velocity-shear rate curve calculation. (B) Cells adhesion curves.
(DOCX)
