# Supplementary material for: Probing the Cytoadherence of Malaria Infected Red Blood Cells under Flow
Source: PLoS One. 2013 May 28;8(5):e64763. doi: 10.1371/journal.pone.0064763 (PMC3665641; doi:10.1371/journal.pone.0064763)
Supplement: Text S1 — Micropipette aspiration experiment. Micropipette aspiration were used to study any possible effects of liphophilic styryl dye, FM® 1–43 dye (Invitrogen) staining on the deformability of iRBCs. (DOCX) [file pone.0064763.s001.docx]

**Text S1**

**Micropipette aspiration experiment**

To study any possible effects of liphophilic styryl dye, FM^®^ 1-43 dye (Invitrogen) staining on the deformability of iRBCs, micropipette aspiration technique[[1](#_ENREF_1)] was used to measure the elastic shear moduli of stained and unstained iRBCs. *Plasmodium falciparum* 3D7 was first cultured and enriched using MACS method. The enriched cells were then stained with 5μg/ml FM^®^ 1-43 dye for half an hour and followed by washing with PBS three times. The micropipettes used had inner diameters of about 2μm. The pressure drop rate of 1 Pa s ^-1^ and a total pressure drop of 100 Pa was applied to aspirate and deform each iRBC. The cell membrane change was monitored using 100X oil immersion objective coupled with a 1.6X magnifier. Sequences of images were recorded using a QColor5 High Resolution Color CCD Digital FireWire Camera (Olympus) and processed by QCapture Pro. 6.0 (Olympus). As shown in Figure S1, when using two-sample student-t test, we found that there were no significant differences when comparing both stages of stained iRBCs with their control groups. As such, the elastic shear moduli of the iRBCs at both the trophozoite stage and the schizont stage do not change when stained with FM^®^ 1-43 dye (Invitrogen).





**Figure S1. Measurements of elastic shear modulus of iRBCs using micropipette aspiration.** Box plot of the elastic shear moduli of iRBCs at the trophozoite stage and the schizont stage when stained FM^®^ 1-43 dye (Invitrogen). Unstained iRBCs served as the control groups. The bottom and top of the box denote the 25^th^ and 75^th^ percentiles of the population, respectively, while the bottom and top whiskers denote 10^th^ and 90^th^ percentiles, respectively. There is no statistical difference (*p* > 0.05) between the same stage cells with and without staining, which indicates that staining with the FM^®^ 1-43 dye does not change the deformability of the cell.

1. Hochmuth RM (2000) Micropipette aspiration of living cells. J Biomech 33: 15-22.
