# Supplementary material for: Probing the Cytoadherence of Malaria Infected Red Blood Cells under Flow
Source: PLoS One. 2013 May 28;8(5):e64763. doi: 10.1371/journal.pone.0064763 (PMC3665641; doi:10.1371/journal.pone.0064763)
Supplement: Text S2 — More detail about model description. (A) Velocity-shear rate curve calculation. (B) Cells adhesion curves. (DOCX) [file pone.0064763.s002.docx]

**Text S2**

**A. Velocity-shear rate curve calculation**

For experimental data fitting, we made use of the model in ref. [[1](#_ENREF_1)] as described below.

In this model, we assume that the contact area between an adherent cell and the substrate surface is characterized by its average length, *a*, and width, *b* (as illustrated in Figure S2) and they are approximately equal to each other (*a* ≈ *b*) as was observed from experiments. The motion of the cell is influenced by a resulting load *F* and torque *M* arising from the shear flow with shear rate S acting on it (Figure 3). As a result, the bonds formed previously between the cell and the substrate surface start to rupture at the trailing edge of the contact area due to the tension, *Q_tot_* acting on it. This tension balances the load *F* and torque *M*. The rupture area is characterized in the model by its average length *c* (Figure S2).





**Figure S2.** The contact and rupture areas in our model are characterized by their average lengths *a* and *c*, and their average width *b* (labeled as Figure 2 in ref.[[1](#_ENREF_1)] ).

To describe the cell motion near the surface, we used a steady-state approximation where we consider the long time average of the cell movement, which is typically studied in experiments. In this case, the cell velocity is mainly determined by the bonds’ rupture rate. Knowing the size of the rupture area *c* and the average lifetime *τ* of a single adhesion site in this area, it is easy to find the average cell velocity *v*:

$v=\frac{c}{\tau}$ (1)

The average lifetime of an adhesion site in the rupture area is a function of the tension *Q* applied to it. This tension is related to the total tension *Q_tot_* experienced by all the adhesion sites in the rupture area through the following equation:

$KQ=Q_{tot}$ (2)

Here, *K* is the total number of adhesion sites in the rupture area. Obviously, *K* = *σbc*, where *σ* is the surface density of adhesion sites at the trailing edge of the contact area. Thus, eq. 2 can be rewritten as follows:

$Q=\frac{Q_{tot}}{\sigma bc}$ (3)

The total tension *Q_tot_* is a function of the flow shear rate *S*. In the simplest case when the cell has approximately the shape of a sphere, this function can be written in an explicit form. Expressing the total tension *Q_tot_* through the load *F* and torque *M* by assuming force equilibrium, one can derive that (see ref. [[1](#_ENREF_1)] for details):

$Q_{tot}\approx4\pi\eta\frac{r^{2}}{a}\left[7.1rS+\left(2.4\ln\left(\delta/r\right)-6.0\right)v\right]$ (4)

Here *r* is the radius of the cell; *δ* is the size of the gap between the rolling cell and the wall (*δ* << *r*) and *η* is the buffer viscosity.

In a general case, each of the cell- surface adhesion sites is formed by a cluster of *N* adhesion proteins. In our model, the surface density *σ* of the adhesion sites in the rupture area was calculated by solving the master equations for a simple reaction *A+B* → *AB* , which describes the binding between adhesion protein clusters *A* on the cell membrane and *B* on the surface:

$\left\{\begin{matrix} \frac{d\sigma_{A}}{dt}=\frac{d\sigma_{B}}{dt}=-k_{+}\sigma_{A}\sigma_{B} \\ \frac{d\sigma_{AB}}{dt}=k_{+}\sigma_{A}\sigma_{B} \end{matrix}\right.$ (5)

with additional constraints:

$\left\{\begin{matrix} \sigma_{A}+\sigma_{AB}=\sigma_{A}^{0} \\ \sigma_{B}+\sigma_{AB}=\sigma_{B}^{0} \end{matrix}\right.$ (6)

Here *σ_A_*, *σ_B_*, *σ_AB_*, are the surface densities of adhesion protein clusters *A*, *B* and adhesion sites *AB* at time *t*, respectively; *σ^0^_A_* and *σ^0^_B_* are the total surface densities of adhesion protein clusters *A* and *B*; *k_+_* is the second order reaction rate for diffusive binding of the protein clusters. Here we assume that each adhesion site has a very small probability to dissociate until it reaches the rupture area where this process is greatly accelerated. This is a reasonable assumption taking into account that every bond in the contact area excluding the rupture area is compressed due to the shear flow pushing the cell against the surface, see Figure 3. The bond compression reduces the dissociation rate for the reverse reaction *AB* → *A+B*, which, therefore, can be neglected.

The final solution of eq. 5 and 6 is:

$\sigma_{AB}\left(\Delta t\right)=\left\{\begin{matrix} \begin{matrix} \frac{k_{+}\sigma_{0}^{2}\Delta t}{1+k_{+}\sigma_{0}\Delta t}, & \sigma_{A}^{0}=\sigma_{B}^{0}=\sigma_{0} \end{matrix} \\ \begin{matrix} \frac{\sigma_{A}^{0}\sigma_{B}^{0}\left[\exp\left(k_{+}M\Delta t\right)-\exp\left(k_{+}m\Delta t\right)\right]}{M\times\exp\left(k_{+}M\Delta t\right)-m\times\exp\left(k_{+}m\Delta t\right)}, & \sigma_{A}^{0}\neq\sigma_{B}^{0} \end{matrix} \end{matrix}\right.$ (7)

Here *M* = max(*σ^0^_A_*; *σ^0^_B_*) and m = min(*σ^0^_A_*; *σ^0^_B_*) are the maximum and minimum of the two values.

In living cells, the rupture area is much smaller than the contact area (i.e. *c* << *a*, see Table 1). Thus, the surface density of the adhesion sites *σ* is approximately the same at any location inside the rupture area since each adhesion protein cluster spends approximately the same amount of time *Δt* = *a/v* in order to move from the leading to the trailing edge of the contact area. Therefore, *σ* can be found as:

$\sigma=\sigma_{AB}\left(\Delta t=a/v\right)$ (8)

Thus, substituting eq. 4, 7 and 8 into eq. 3, one can find the tension *Q* acting on a single adhesion site in the rupture area. Knowing this force, it is possible to calculate the average life time of an adhesion site *τ* using a simple kinetic scheme depicted in Figure S3.





**Figure S3.** Kinetic model, which was used for calculation of the average lifetime τ of a single adhesion site.

Under the tension *Q* acting on the adhesion site, the bonds composing it start to rupture one by one until all of them are broken. Therefore, each physical state of the adhesion site can be represented by the number of bonds – from 0 to *N* as shown in Figure S3. Transitions between these states can be described by rates *k_i,i+1_* and *k_i,i-1_* (0 ≤ *i–1, i, i+1* ≤ *N*) of a single bond formation and rupture, respectively. Transition 0→1 in the general case can be neglected since right after the rupture of the final bond (transition 1→0), the two protein clusters which formed the adhesion site previously are quickly separated by a large distance. Bond formation rates *k_i,i+1_* are assumed to be independent from the tension as in ref.[[2](#_ENREF_2)], while for the bonds dissociation rates *k_i,i-1_*, we used the Bell-Evans model[[3](#_ENREF_3)]:

$\left\{\begin{matrix} k_{i,i-1}=ik_{off}e^{Qx^{\#}/ik_{B}T} \\ k_{i,i+1}=\left(N-i\right)^{2}k_{on} \end{matrix}\right.$ (9)

Here *x^#^* is the binding potential width; *k_on_* is the rate of a single bond formation between two proteins in the adhesion site (if *N* > 1); *k_off_* is the unstressed rate of a single bond dissociating; *k_B_* is Boltzmann constant and *T* is temperature. In eq. 9, we assume that the tension *Q* in each state *i* is equally distributed between the *i* bonds. Also, here we take into account the fact that if there are *(N–i)* unbound proteins in each cluster in the adhesion site, then the total number of possible ways to form a new bond is *(N–i)^2^*.

Using the formula of the mean turnover time for chain reactions from ref. [[4](#_ENREF_4),[5](#_ENREF_5)], the average lifetime *τ* of a single adhesion site consisting from *N* adhesion proteins (in each protein cluster forming the adhesion site) can be found from the following two equations:

$\tau\left(N\right)=\sum_{i=1}^{N} \rho_{i}+\sum_{j=1}^{N-1} \left[\frac{1}{\rho_{j}k_{j,j+1}}\sum_{m=j+1}^{N} \rho_{m}\right]$ (10)

where:

$\rho_{1}=\frac{1}{k_{1,0}}$ and $\rho_{i}=\prod_{j=1}^{i-1} k_{j,j+1}/\prod_{j=1}^{i} k_{j,j-1}$ (11)

Thus, the final algorithm of the velocity-shear rate, *v-S*, curve calculation is as follows:

1. Find the surface density of adhesion sites, *σ*, in the rupture area from eq. 7 and 8..
2. Calculate the total tension *Q_tot_* acting on adhesion sites in the rupture area using eq. 4.
3. Find the average tension *Q* acting on a single adhesion site from eq. 3.
4. Calculate the average lifetime of a single adhesion site in the rupture area from eq. 9-11, and
5. Calculate the steady-state cell velocity *v* as a function of shear rate *S* using eq. 1.

**B. Cells adhesion curves**

We have found that as was observed in ref. [[1](#_ENREF_1)], the velocity-shear rate curve, *v-S*, has a bistable behavior (see, for example, Figure 4A). The minimum of this curve determines the catching efficiency of adhesion proteins on the cell surface – if the flow shear rate is below the shear rate corresponding to the minimum, then the cell will adhere to the surface. Otherwise, it will keep moving with a high velocity without stable bonds formation with the surface. Whereas, the curve maximum characterizes the strength of adhesion bonds – adherent cell will not detach from the surface unless the shear rate is higher than the shear rate corresponding to the curve maximum.

Variation of the model parameters in our calculations has shown that the positions of the *v-S* curve extremums are most sensitive to the following parameters: 1) the contact and rupture area’s lengths and width, *a*, *b* and *c, respectively*; 2) bond unstressed rupture rate *k_off_*, and binding potential half-width *x*^#^; 3) the minimal of the total concentrations of adhesion proteins on the cell and coverslip surfaces, min(*σ*_1_*=N*×*σ^0^_A_*; *σ*_2_=*N*×*σ^0^_B_*). Also, the model shows a sensitivity to the adhesion sites formation rate, *k*_+_, unless it has a relatively high value (*k*_+_ >> *v*/[*a*×min(*σ^0^_A_*; *σ^0^_B_*)]) at which the surface density of adhesion sites in the rupture area saturates (*σ* ≈ min(*σ^0^_A_*; *σ^0^_B_*), as can be seen from eq. 7). Interestingly, from our calculations we have found that the extreme shear rates weakly depend on the average number of proteins in a single adhesion site *N*, and a single bond formation rate *k_on_*.

From our experimental measurements, we know that iRBCs were not identical to each other and the contact area size varied considerably from one cell to another. Thus, for the fitting of the adhesion curves (Figure 7A-C) it is important to know the standard deviations of the contact area length and width, *δa* and *δb*, since these two parameters determine the gradient of the adhesion curves’ slopes (for details, see discussion in ref. [[1](#_ENREF_1)]). Substituting the experimentally measured variables (*a*, *δa*, *b*, *δb*, *k_off_*, *x*^#^ and the knobs concentration on the iRBCs surface, *σ^0^_A_*) into the model and computationally generating a cell population with experimentally determined contact area size distribution (*a*±*δa*, *b*±*δb*), we calculated the percentage of attached cells at a given shear rate *S*_given_, as follows:

1. For each cell from the generated population, we plotted velocity-shear rate *v-S* curve and determined the shear rate at its maximum and minimum, *S*_max_ and *S*_min_.
2. For the increasing shear rate detachment curve, we compared S_given_ with S_max_ – according to our model, if *S*_given_ ≤ S_max_ then the cell was still attached to the surface, otherwise it was assumed to be detached. For the decreasing shear rate curve, the idea is the same except that *S*_given_ s compared to *S*_min_ instead.
3. By conducting steps a and b for every cell from the population, it would be easy then to work out the percentage of cells adherent to the surface.

By performing experiments at different concentrations of the ligand (CD36) and fitting the experimental detachment curves to the measured data, we found the values of unknown variables (*c*, *k*_+_, *N* and *σ^0^_B_*) as reported in the main text.

1. Efremov A, Cao JS (2011) Bistability of Cell Adhesion in Shear Flow. Biophysical Journal 101: 1032-1040.

2. Erdmann T, Schwarz US (2004) Stability of adhesion clusters under constant force. Physical Review Letters 92.

3. Bell GI (1978) Models for the specific adhesion of cells to cells. Science 200: 618-627.

4. Bar-Haim A, Klafter J (1998) On mean residence and first passage times in finite one-dimensional systems. Journal of Chemical Physics 109: 5187-5193.

5. Cao JS, Silbey RJ (2008) Generic Schemes for Single-Molecule Kinetics. 1: Self-Consistent Pathway Solutions for Renewal Processes. Journal of Physical Chemistry B 112: 12867-12880.
